# Does an app make patients happy? Impact of a novel medical history app on patient satisfaction in urgent care consultations in Germany: cluster-randomized interventional trial ‘DASI’

**DOI:** 10.1186/s12913-026-14795-6

**Published:** 2026-05-29

**Authors:** Eva Maria Noack, Kai Antweiler, Frank Müller, Dominik Schröder, Tim Friede, Eva Hummers, Lea Roddewig

**Affiliations:** 1 Department of General Practice, University Medical Center Göttingen, Göttingen, Lower Saxony, Germany; 2 Department of Medical Statistics, University Medical Center Göttingen, Göttingen, Lower Saxony, Germany; 3 Department of Family Medicine, Michigan State University, Grand Rapids, Michigan, USA

**Keywords:** Patient satisfaction, Digital health, Medical history taking, Out-of-hours care, EUROPEP, Healthcare innovation

## Abstract

**Background:**

Patient satisfaction is an important indicator of healthcare quality. However, time constraints in primary care limit effective communication and history-taking. Digital medical history systems have the potential to improve the quality of medical consultations by enabling patient preparation and providing physicians with comprehensive pre-consultation information. This study aimed to evaluate the impact of a novel medical history-taking app (DASI app) on patient satisfaction in out-of-hours practices (OOHP) in Germany.

**Methods:**

We conducted a two-center, cluster-randomized trial over 12 months. Within each practice, weeks were randomized to either an intervention or control group, resulting in a cluster-randomized trial (CRT) with clustering in weeks within the same practice. Patients either used the DASI app before consultations (intervention group) or received standard care (control group). The DASI app is a patient-facing tool that guides patients through a dynamic questionnaire adapted to the selected complaints and previous answers. Patient satisfaction was measured using 17 items from the EUROPEP instrument covering relation and communication, medical care, and information and support. Additionally, we collected sociodemographic data. Statistical analyses included Mann-Whitney *U* tests for individual items and t-tests for domain scores. Analyses were performed with R, version 4.5.2.

**Results:**

Among 1,460 approached patients, 1,040 (71%) were enrolled and 1,034 included in analyses. Patient median age was 31 years, with 60% female participants. Patients in the intervention group (*n* = 496) showed significantly better ratings in 12 of 17 EUROPEP items compared to controls (*n* = 538) and demonstrated significantly higher satisfaction across all three EUROPEP domains. Between 78 and 96% of patients rated care as excellent or very good, with intervention group patients more likely to select top-level evaluations in 9 of 17 items.

**Conclusions:**

Digital medical history taking significantly enhanced patient satisfaction in urgent care settings. The app likely improved satisfaction through multiple pathways including increased patient empowerment, better consultation preparation, and more efficient physician-patient interactions. These findings demonstrate that user-friendly digital tools can meaningfully enhance patient experience without disrupting existing workflows, supporting healthcare digitalization efforts while maintaining high-quality patient-centered care.

**Trail registration:**

German register for clinical trials (DRKS00026659; date of registration: 2021-11-03).

**Supplementary Information:**

The online version contains supplementary material available at 10.1186/s12913-026-14795-6.

## Background

Patient satisfaction is a key indicator of healthcare quality [1]. High levels of patient satisfaction are crucial for establishing trust and a positive relationships between patients and healthcare professionals: Satisfied patients are more likely to openly share their health concerns and adhere to treatment recommendations [2–4]. They are more likely to actively participate in their care and less likely to switch providers [5]. This positive dynamic contributes to better health outcomes and a more efficient healthcare system as a systematic review showed that clinical and quality outcomes are positively influenced by patient experiences with health care [6].

Patient satisfaction is influenced by various factors, including communication, consultation time, and efficient medical history taking [7–10]. In Germany, the average primary care consultation lasts only 7.6 min [11]. This time includes taking the medical history, performing a physical examination and discussing possible therapies. Limited time for efficient history taking and effective communication is thus a major issue in medical care in Germany with a potential impact on patient satisfaction. The anticipated shortage of physicians [12, 13] will likely increase the time constraints for consultations. This situation underscores the need to optimize the patient encounter process without compromising satisfaction. Digital medical history systems offer a potential solution by empowering patients to actively participate in their care and providing physicians with comprehensive information prior to a face-to-face consultation. Physicians can then provide more consultation time, devote their full attention to patients’ concerns and facilitate more in-depth discussions with them. The review by Jimenez et al. described the impact of digital medical history systems on the consultation but patient satisfaction was not yet assessed [14].

In this study, we evaluate the impact of a novel app (“DASI app”) for medical history taking in general practice on patient satisfaction in out-of-hours practices (OOHP) in Germany. Our aim is to determine whether using the app prior to medical consultations enhances patient satisfaction. The app’s usability and the quality of the collected information was previously assessed [15–17].

## Methods

### Study design

**Fig. 1:**
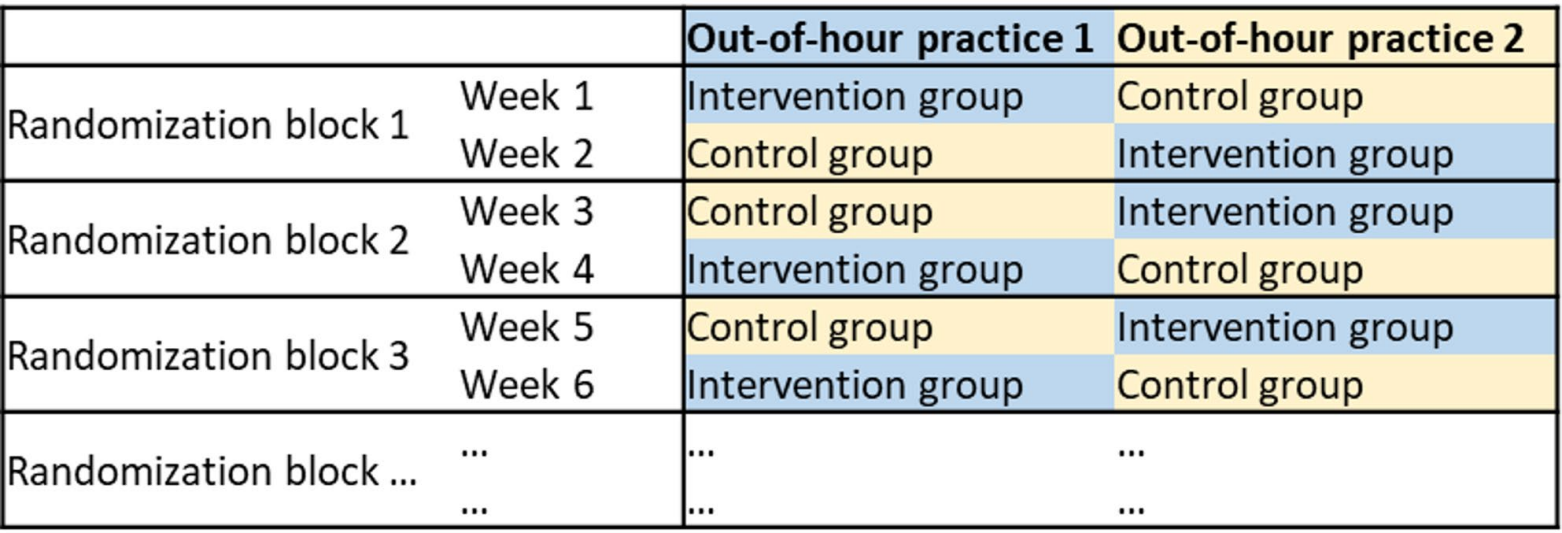
Example of two-week block randomization of participating out-of-hours practices for the first 6 weeks

This paper reports on a secondary outcome of a two-center, unblinded, cluster-randomized trial (CRT) conducted over 12 consecutive months, with the primary outcome being diagnostic accuracy. The study procedures have been outlined in a study protocol [18]. In brief, we implemented the DASI app in two out-of-hours practices (OOHP) in Germany. In the intervention group, patients used the app to take their medical history before consultations and the obtained information was transferred to their electronic medical record (EMR) for physicians to review beforehand. In the control group, patients did not use the app before consultations, no prior information was thus available to physicians. For randomization, we divided the study period into one-week blocks, randomly assigning each block to either intervention or control group. In each week, one practice was assigned to the intervention group, the other practice in the control group. Thus, all patients seen in a particular practice within a given week were part of the same group. We ensured that no practice remained in the same group for more than two consecutive weeks by using two-week blocks for randomization (as illustrated in Fig. 1).

A member of the study team not involved in data collection generated the random allocation sequence. The recruitment period spanned from March 1, 2022, to February 28, 2023. The study was open-label, meaning that both patients and treating physicians were aware of the assigned group.

Institutional Review Board of the University Medical Center Göttingen approved the study (approval number 26/3/21). The study was registered with the German Clinical Trials Register (DRKS00026659; date of registration: November, 3rd 2021) and was conducted in accordance with the Declaration of Helsinki. We follow the CONSORT 2010 statement for reporting cluster-randomized trials [19].

### Setting

The study was conducted in two OOHP in Lower Saxony, Germany. These practices are staffed by rotating physicians from practices within the district, representing various specialties (e.g., internal medicine, dermatology, gynecology and obstetrics, otolaryngology, and neurology) and experience levels. OOHP operate outside regular physicians’ working hours, i.e. in the evenings, on Wednesday and Friday afternoons, on weekends, and on public holidays, to provide medical care to patients with acute but not life-threatening conditions outside regular working hours. They serve a diverse patient population with varying health needs, mirroring those found in general practices. However, unlike general practices, where physicians often have established relationships with patients, OOHP physicians mostly encounter new patients. Lacking access to shared patient files, physicians must gather more information to construct a comprehensive medical history, and patients may also be more inclined to provide detailed information, which can be a challenging task given the high workload and brief consultation times in OOHP [20].

The two OOHPs were purposively selected to represent different healthcare environments while addressing constraints during the COVID-19 pandemic (e.g. travel restrictions). One OOHP was located in Göttingen, a university city with high proportion of university students, thus serving a rather young urban population. The other OOHP was located in Northeim, a small town with a rural catchment area of small villages with various demographic characteristics. Both OOHP were in proximity to the conducting department. This selection allowed for comparison between different practice settings with the practical constraints imposed by pandemic-related restrictions.

### Intervention

The DASI-app is a patient-facing tool, used by patients in the waiting room before a physician encounter. It evolved from a multilingual medical history tool used in health care for migrants and refugees [21, 22]. For this project, it was adapted for the study setting. After providing basic demographic information, patients can choose from a list of common acute health concerns that are typical of primary care consultations and receive follow-up questions tailored to their specific condition. Depending on the condition, the app also inquires about pre-existing conditions, medications, lifestyle habits, and family health history. To ensure accessibility, the app uses plain language. A previous study confirmed that the app is user-friendly and does not require prior instruction [16]. Depending on the number of initially chosen complaints and on the number of follow-up questions, data entry takes 5–15 min. Once completed, the app generates a structured medical history report, which can be transferred to the patient’s EMR for physician review. The app is available in multiple languages; for this study, we used the German version. As a web app, the DASI app can be used on different devices and could be used by patients from home; in this study, patients used the app on a study tablet (iPad Mini 5; Apple Inc., Cupertino, CA, USA) in the OOHP.

### Study participation: inclusion and exclusion criteria

Potential study participants were informed about the study by trained study nurses who checked the study’s eligibility criteria. To be eligible, patients had to be (a) seeking care at one of the participating OOHP for an acute condition, (b) at least 18 years old (legal of age in Germany), and (c) able to provide written informed consent. Conversely, patients were excluded from the study if they were (a) under 18 years old, (b) experiencing a medical emergency, (c) requiring immediate medical attention, and (d) unable to provide informed consent due limited German language proficiency or other reasons. Participation in the study was voluntary. Participants were required to sign a written informed consent form before participating in the study. Patients were free to withdraw from the study at any time without providing a reason.

### Measures

Patients’ overall satisfaction with the consultation was assessed using questions from the standardized and validated questionnaire European Project on Patient Evaluation of General Practice Care Questionnaire (EUROPEP) in German language [23–25]. This instrument comprises items on the quality of care in General Practice, including the domains (1) relation and communication, (2) medical care (3) information and support, (4) continuity and co-operation, and (5) facilities, availability, and accessibility. Participants rate the items on a five-point Likert scale ranging from “excellent” (1) to “poor” (5). Patients can select the response option “not applicable” if items do not apply to them. For this study, we used the revised version EPA HA 3.2 as provided by the aQua Institute (Institut für angewandte Qualitätsförderung und Forschung im Gesundheitswesen GmbH, Maschmühlenweg 8–10, 37073 Göttingen, www.aqua-institut.de). We deliberately selected 17 items to be included in the study (items# 1–5, 8–9, 11–16, 18–20, 22) that relate to the General Practitioner’s (GP’s) performance but not to the practice (e.g. organizations of care, arrangement of appointments) as these questions to not apply for the OOPH setting. We further adapted the introductory wording to fit the setting of an OOHP, i.e. patients were asked to rate the preceding consultation but not their experience over the preceding year, as most patients do not visit the OOHP regularly. We changed item #17 to use gender-inclusive language. The use and adaptation of the EUROPEP questionnaire was approved by the aQua Institute. The German version of the EUROPEP items used in this study and an English forward-translation are provided in Additional file 1.

### Data collection

Following enrollment, intervention group patients received a study tablet (iPad mini 5; Apple Inc., Cuppertino CA) with the DASI-app. They completed the medical history query with the DASI-app prior to the encounter. This included a question on the severity of complaints. The report was transferred to the patient’s EMR. Following the consultation, patients completed the EUROPEP and a short sociodemographic survey. This survey was conducted via LimeSurvey and displayed on the study tablets.

**Fig. 2:**
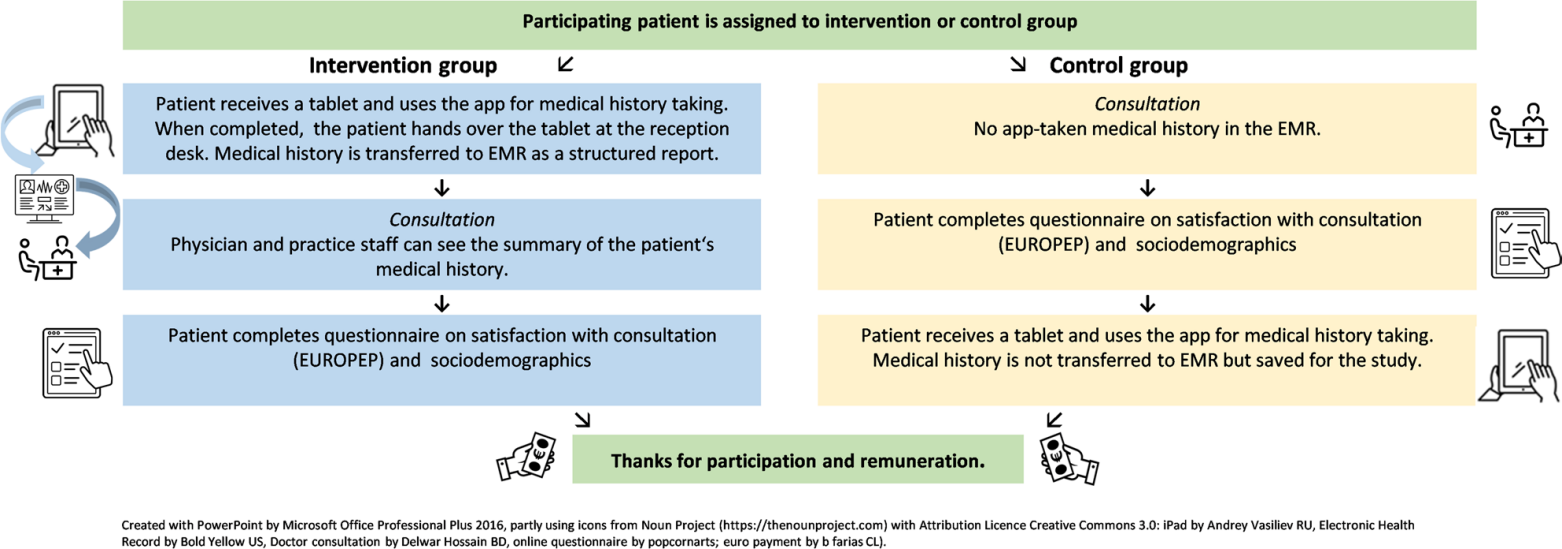
Process of data collection. Abbreviations: EMR: electronic medical record; EUROPEP: European Project on Patient Evaluation of General Practice Care Questionnaire

Patients in the control group proceeded to the consultation first. After the consultation they completed the EUROPEP and sociodemographics survey before using the DASI-app to prevent any potential bias from comparing the doctor’s consultation to the DASI-app. Participants received an expense allowance of 15 Euro. Figure 2 shows the process of data collection.

### Statistical analysis

All hypothesis tests contrasted either the intervention with the control group or the two study sites. Except for baseline characteristics, all tests were adjusted with mixed models for the severity of complains (numerical severity score as a fixed effect) and the temporal clustering (as a random effect). Two-sided p values < 0.05 were interpreted as statistically significant. Analyses were performed with R, version 4.5.2 [26].

#### Baseline characteristics

Characteristics of participants (Table 1) were compared between groups by nonparametric tests: Categorical variables are summarized as absolute and relative frequencies (n, %); group differences were examined with the χ² test. Age, and “number of complaints selected in the app” are reported as median and interquartile range (IQR) and between-group comparisons were performed with the Mann–Whitney *U* test. When the two groups shared an identical median, the mean ± standard deviation (SD) and the result of a two-sample t-test are additionally provided. “Severity of complaints” was treated as an ordinal variable. Its categories are presented as n (%) and compared with the Mann–Whitney U test.

#### EUROPEP item comparisons

For each individual EUROPEP item, responses were summarized by median (Q25-Q75), mean (SD), n (%) and compared between groups with linear mixed models. The response option “do not know/not applicable” was regarded as a missing value (missing values (n, %) for each item are reported in Additional file 3).

In an additional analysis, each EUROPEP item was dichotomized into “top-box” ratings (1 or 2) versus all other ratings (3–5). Results are shown as n (%) and compared between groups with logistic mixed models.

Because of the high percentage of university students in the OOHP in Göttingen compared to Northeim, we carried out additional analyses for the study sites excluding university students.

#### EUROPEP domain score analysis

EUROPEP domain scores were calculated as the arithmetic mean of the non-missing items in each domain: relation & communication (items 1–5), medical care (items 6, 7, 12, 13), and information & support (items 8–11, 14–17). Domain scores are presented as mean with 95% confidence intervals (CI) and compared between groups using linear mixed models.

## Results

### Sample characteristics

**Fig. 3:**
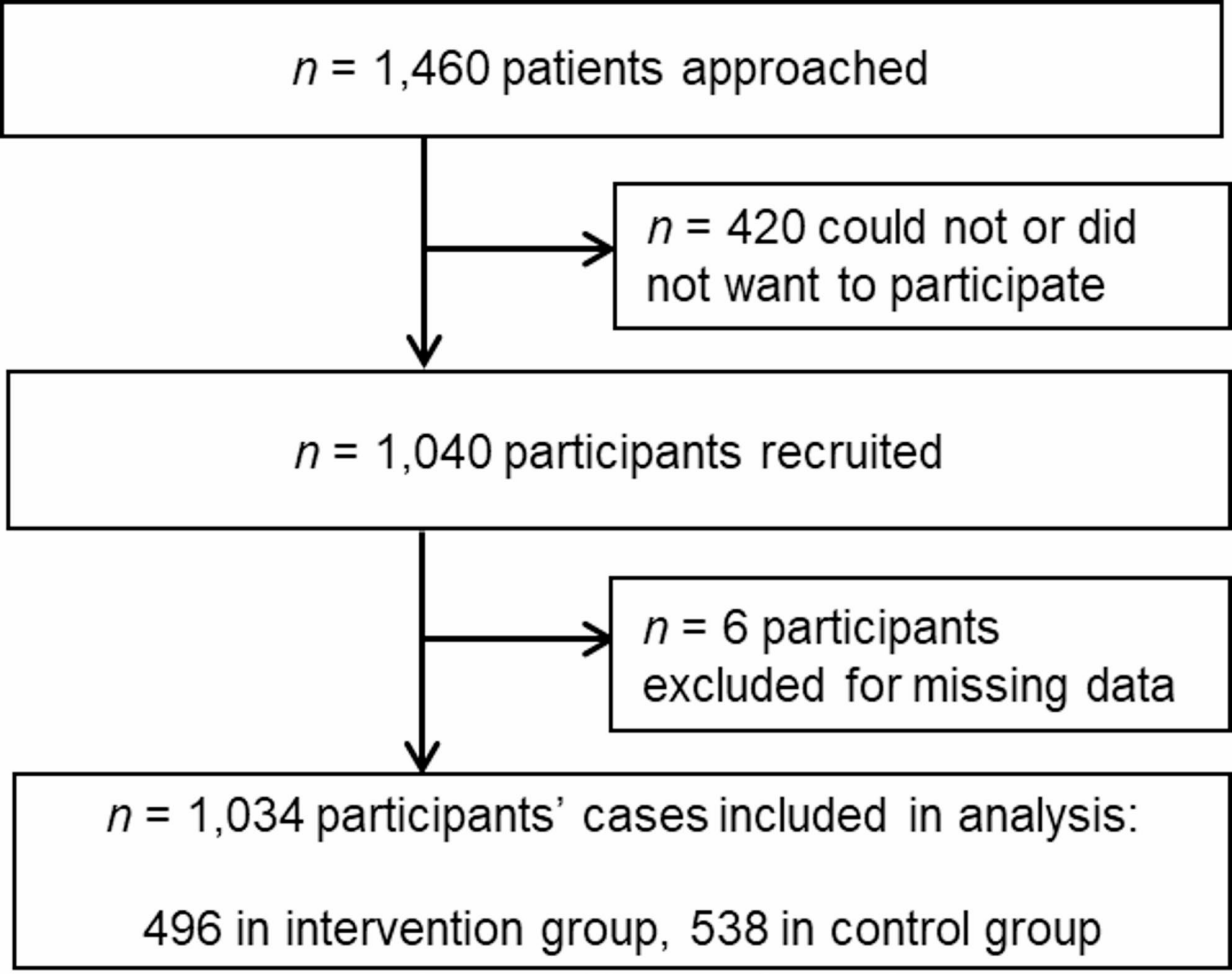
Flowchart

From 1,460 approached patients 1,040 patients (71%) were recruited for the study. In 4 cases the app was not completed after the consultation, in 2 cases data from the EUROPEP was missing. Data from 1,034 participants were included in the analyses. Figure 3 shows the flowchart and Table 1 shows characteristics of included patients.

The median age of participating patients was 31 years (IQR 24, 44) years, 40% being male and 60% female. On average, patients had one complaint (IQR 1, 2). Participants in the intervention group were older (median 32 years (IQR 25, 46) vs. 30 years (IQR 23, 43), *p* = 0.022), reported more complaints (mean 1.88 (SD 1.60) vs. 1.62 (SD 1.26), *p* = 0.004) and rated their complaint more severe (*p* = 0.01) in the app.

**Table 1:** Characteristics of participants - comparison between intervention and control group

		All *n* = 1,034	Control group *n* = 538	Intervention group *n* = 496	*p*
Sex, n (%)	male	410 (40%)	218 (41%)	192 (39%)	0.552
	female	624 (60%)	320 (59%)	304 (61%)	
Age, median (IQR)	years	31 (24, 44)	30 (23, 43)	32 (25, 46)	**0.022**
Number of complaints selected in the app, median (IQR)	n	1 (1, 2)	1 (1, 2)	1 (1, 2)	**0.016**
Severity of complaints, n (%)	I don’t feel sick	112 (11%)	63 (12%)	49 (9.9%)	0.01
	Mild	192 (19%)	107 (20%)	85 (17%)	
	Medium	512 (50%)	272 (51%)	240 (48%)	
	Severe	195 (19%)	85 (16%)	110 (22%)	
	Unbearable	20 (1.9%)	9 (1.7%)	11 (2.2%)	
Highest professional qualification, n (%)	Master’s degree/Diploma/State examination/ PhD	140 (14%)	69 (13%)	71 (14%)	0.189
	Bachelor‘s degree	82 (8.0%)	49 (9.1%)	33 (6.7%)	
	Master craftsman/technician or equivalent	42 (4.1%)	22 (4.1%)	20 (4.1%)	
	Completed vocational training	248 (24%)	128 (24%)	120 (24%)	
	High school diploma / Advanced technical college certificate	224 (22%)	120 (22%)	104 (21%)	
	Secondary school certificate	177 (17%)	101 (19%)	76 (15%)	
	Elementary/lower secondary school certificate	88 (8.6%)	35 (6.5%)	53 (11%)	
	Other qualifications	14 (1.4%)	7 (1.3%)	7 (1.4%)	
	No degree	14 (1.4%)	5 (0.9%)	9 (1.8%)	
Current employment status, n (%)	Employed/working	563 (56%)	295 (56%)	268 (56%)	0.347
	In vocational training	85 (8.4%)	43 (8.1%)	42 (8.8%)	
	University student	160 (16%)	94 (18%)	66 (14%)	
	Voluntary/civilian/military service	6 (0.6%)	5 (0.9%)	1 (0.2%)	
	Student at school	24 (2.4%)	12 (2.3%)	12 (2.5%)	
	Retired	63 (6.2%)	29 (5.5%)	34 (7.1%)	
	Unemployed	20 (2.0%)	11 (2.1%)	9 (1.9%)	
	other	88 (8.7%)	40 (7.6%)	48 (10%)	
German as native language, n (%)	yes	906 (88%)	479 (89%)	427 (87%)	0.202
Study center, n (%)	Northeim	461 (45%)	227 (42%)	234 (47%)	0.122

P-values < 0.05 in bold (Mann-Whitney *U*-Test for continuous variables, Chi-square test for categorical variables, Mann–Whitney *U* test for ordinal variables)

Participants recruited in Göttingen were younger than in Northeim (median 29 years vs. 34 years, *p* < 0.001). There were also significant differences regarding participant’s highest professional qualification and main employment status with higher degrees in Göttingen and more university students (27% vs. 2.2%, *p* < 0.001) (Additional file 2).

### Satisfaction with consultation

Overall, study participants were very positive about the care provided in the OOHP. For most of the EUROPEP questionnaire items more than half of the participants rated care as ‘excellent’, on the five-point Likert scale. Best-rated items were item 5 (‘listening to you’) with a mean score of 1.27 and item 3 (‘making it easy to tell about the problem’) with a mean score of 1.34. Worst rated item was item 10 (‘inform about the effects and possible side effects of the medication’) with a mean score of 1.81 (see Table 2). Additional file 3 shows the results of all response options for all items for intervention and control group. Items that were not relevant to respondents were marked as “not applicable”. In Northeim, 15 of 17 items were evaluated significantly more positively than in Göttingen (see Additional file 4). A subgroup analysis shows that when excluding university students, only in 3 of 17 items evaluations differ significantly between centers (see Additional file 4).

### Satisfaction with consultation by group

Patients in the intervention group showed significantly better ratings in 12 of 17 items see (see Table 2). For all dimensions, patients in the intervention group scored significantly lower scores indicating higher satisfaction compared to controls (relation and communication *p* = 0.003, medical care *p* < 0.001, information and support *p* < 0.001) (see Additional file 5).

**Table 2:** EUROPEP – comparison between intervention and control group

EUROPEP Item			All *N* = 1,034		Control group *n* = 538		Intervention group *n* = 496		
Nr.	What is your view on the GP with respect to…?	n	Median (IQR)	*M* (SD)	Median (IQR)	*M* (SD)	Median (IQR)	*M* (SD)	p (adj)*
1	…making you feel you had time during consultation?	1,033	1.00 (1.00, 2.00)	1.37 (0.68)	1.00 (1.00, 2.00)	1.43 (0.76)	1.00 (1.00, 1.00)	1.30 (0.59)	**0.002**
2	…interest in your personal situation?	1,026	1.00 (1.00, 2.00)	1.43 (0.73)	1.00 (1.00, 2.00)	1.47 (0.77)	1.00 (1.00, 2.00)	1.38 (0.67)	**0.032**
3	…making it easy for you to tell him or her about your problems?	1,027	1.00 (1.00, 2.00)	1.34 (0.66)	1.00 (1.00, 2.00)	1.38 (0.69)	1.00 (1.00, 1.00)	1.29 (0.61)	**0.018**
4	…involving you in decisions about your medical care?	983	1.00 (1.00, 2.00)	1.49 (0.78)	1.00 (1.00, 2.00)	1.56 (0.83)	1.00 (1.00, 2.00)	1.41 (0.72)	**0.001**
5	…listening to you?	1,028	1.00 (1.00, 1.00)	1.27 (0.60)	1.00 (1.00, 1.00)	1.30 (0.63)	1.00 (1.00, 1.00)	1.25 (0.56)	**0.139**
6	…thoroughness?	1,024	1.00 (1.00, 2.00)	1.47 (0.79)	1.00 (1.00, 2.00)	1.55 (0.84)	1.00 (1.00, 2.00)	1.38 (0.72)	**< 0.001**
7	…physical examination of you?	890	1.00 (1.00, 2.00)	1.49 (0.76)	1.00 (1.00, 2.00)	1.53 (0.77)	1.00 (1.00, 2.00)	1.44 (0.75)	0.069
8	…explaining the purpose of tests and treatments?	910	1.00 (1.00, 2.00)	1.55 (0.83)	1.00 (1.00, 2.00)	1.64 (0.89)	1.00 (1.00, 2.00)	1.46 (0.75)	**0.001**
9	…explaining you advantages and disadvantages of treatment possibilities?	722	1.00 (1.00, 2.00)	1.72 (0.96)	2.00 (1.00, 2.00)	1.82 (1.00)	1.00 (1.00, 2.00)	1.63 (0.90)	**0.005**
10	…inform you about the effects and possible side effects of the medication he/she prescribed?	676	1.00 (1.00, 2.00)	1.81 (1.04)	2.00 (1.00, 3.00)	1.95 (1.12)	1.00 (1.00, 2.00)	1.67 (0.93)	**< 0.001**
11	…telling you what you wanted to know about your symptoms and/or illness?	924	1.00 (1.00, 2.00)	1.53 (0.82)	1.00 (1.00, 2.00)	1.58 (0.85)	1.00 (1.00, 2.00)	1.48 (0.79)	**0.046**
12	…inform you about the pain you could expect during the examination and treatment?	650	1.00 (1.00, 2.00)	1.60 (0.90)	1.00 (1.00, 2.00)	1.67 (0.97)	1.00 (1.00, 2.00)	1.53 (0.81)	**0.037**
13	…ask you about any pain?	978	1.00 (1.00, 2.00)	1.39 (0.79)	1.00 (1.00, 2.00)	1.46 (0.86)	1.00 (1.00, 1.00)	1.32 (0.71)	**0.004**
14	…helping you deal with emotional problems related to your health status?	820	1.00 (1.00, 2.00)	1.63 (0.95)	1.00 (1.00, 2.00)	1.72 (1.01)	1.00 (1.00, 2.00)	1.55 (0.87)	**0.005**
15	…helping you understand the importance of following his or her advice?	775	1.00 (1.00, 2.00)	1.62 (0.90)	1.00 (1.00, 2.00)	1.69 (0.98)	1.00 (1.00, 2.00)	1.54 (0.82)	**0.019**
16	…inform you about what you can do yourself to heal/improve your symptoms (e.g. in everyday life)?	833	1.00 (1.00, 2.00)	1.58 (0.95)	1.00 (1.00, 2.00)	1.64 (0.99)	1.00 (1.00, 2.00)	1.51 (0.90)	**0.023**
17	…preparing you for what to expect from specialist or hospital care?	606	1.00 (1.00, 2.00)	1.59 (0.87)	1.00 (1.00, 2.00)	1.65 (0.92)	1.00 (1.00, 2.00)	1.52 (0.80)	**0.04**

Items are scored 1–5 where higher scores represent better experiences. The response scale for all items was a five-point scale with the endpoints very dissatisfied and very satisfied. IQR: interquartile range; *M* arithmetic mean. P-values < 0.05 in bold. *p (adjusted) for severity of complaints and time clusters

Between 78% and 96% of patients scored the single items on the two top level evaluations, i.e. 1 (excellent) or 2 (see Table 3). In 9 from 17 items, patients in the intervention group were more likely to select the most favorable response options 1 (“excellent”) or 2.

**Table 3:** EUROPEP – percentage of patients selecting the two top level evaluations - comparison between intervention and control group

EUROPEP Item			Overall *N* = 1,034	Control group *n* = 538	Intervention group *n* = 496	
Nr.	What is your view on the GP with respect to…?	n	n (%)	n (%)	n (%)	p (adj)*
1	…making you feel you had time during consultation?	1,033	965 (93%)	491 (91%)	474 (96%)	**0.006**
2	…interest in your personal situation?	1,026	948 (92%)	485 (91%)	463 (94%)	0.069
3	…making it easy for you to tell him or her about your problems?	1,027	961 (94%)	494 (93%)	467 (95%)	0.105
4	…involving you in decisions about your medical care?	983	888 (90%)	442 (88%)	446 (93%)	**0.001**
5	…listening to you?	1,028	985 (96%)	507 (95%)	478 (97%)	0.055
6	…thoroughness?	1,024	929 (91%)	473 (89%)	456 (93%)	**0.018**
7	…physical examination of you?	890	799 (90%)	403 (89%)	396 (90%)	0.495
8	…explaining the purpose of tests and treatments?	910	802 (88%)	393 (85%)	409 (91%)	0.002
9	…explaining you advantages and disadvantages of treatment possibilities?	722	582 (81%)	283 (78%)	299 (84%)	**0.021**
10	…inform you about the effects and possible side effects of the medication he/she prescribed?	676	527 (78%)	249 (73%)	278 (83%)	**0.001**
11	…telling you what you wanted to know about your symptoms and/or illness?	924	824 (89%)	420 (88%)	404 (91%)	**0.152**
12	…inform you about the pain you could expect during the examination and treatment?	650	560 (86%)	287 (85%)	273 (88%)	**0.307**
13	…ask you about any pain?	978	899 (92%)	451 (90%)	448 (94%)	**0.023**
14	…helping you deal with emotional problems related to your health status?	820	701 (85%)	340 (83%)	361 (88%)	**0.01**
15	…helping you understand the importance of following his or her advice?	775	654 (84%)	323 (81%)	331 (88%)	**0.014**
16	…inform you about what you can do yourself to heal/improve your symptoms (e.g. in everyday life)?	833	724 (87%)	373 (85%)	351 (89%)	**0.036**
17	…preparing you for what to expect from specialist or hospital care?	606	527 (87%)	263 (85%)	264 (89%)	0.206

P-values < 0.05 in bold. *p (adjusted) for severity of complaints and time clusters

## Discussion

Our study shows, that the use of an app for medical history taking prior to medical consultations in OOHP increases patient satisfaction. In general, patients visiting one of the OOHP were very positive about the care provided. Especially items regarding doctor-patient relation and communication were evaluated positively. In agreement with earlier studies using the EUROPEP [27], we found a strong ceiling effect, i.e. a very high percentage of patients ticking the most favorable response option for almost all items. Even so, the study revealed significant differences in the evaluations between intervention and control group and between the two OOHP. Patients were relatively negative about aspects of care related to information and support. Interestingly, most of the items in this domain were rated better by patients in the intervention group, even though the app did not provide any information on tests, treatments, medication etc. Most of the differences in satisfaction between centers disappear if university students are excluded from analysis. Students are younger in age and young adults have been found to report more negative satisfaction [28, 29].

Former studies on patient satisfaction have mostly dealt with non-modifiable patients’ characteristics (e.g. age, condition, health status, level of education) or features of physicians or practices. Digital innovations in healthcare are often associated with increased time or cost efficiency and improved consultation outcomes [14, 30] but more rarely with patient satisfaction. Studies on the impact of digital innovative approaches on the enhancement of patient experience have focused on telemedicine and mHealth solutions [31, 32]. A qualitative interview study in Sweden found that patients that tested a chat-based and automated medical history-taking service were positive about the tools [33]. To our knowledge, our study is the first clinical study that evaluates the impact of a digital medical history system on patient satisfaction.

**Fig. 4:**
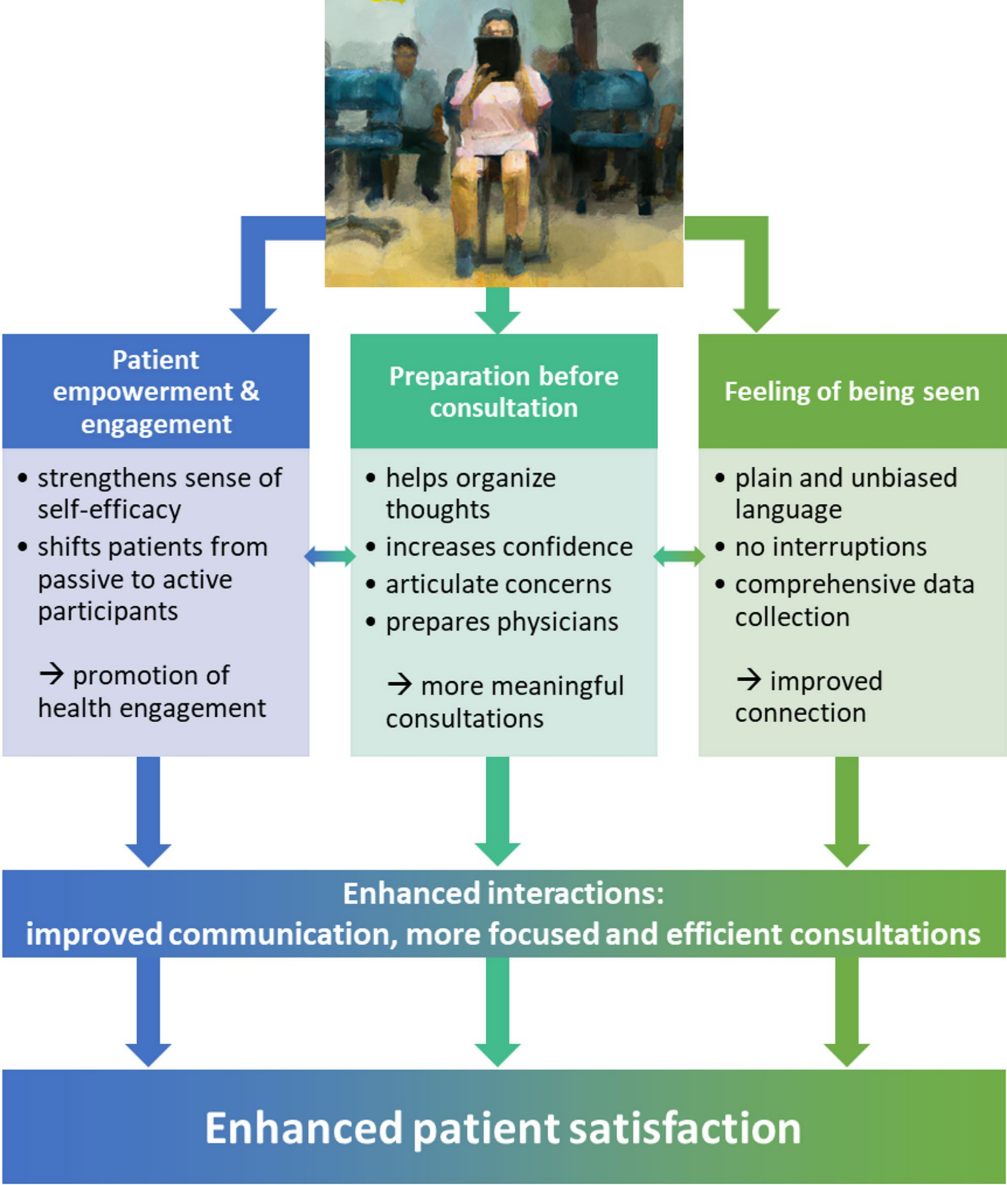
Diagram visualizing potential pathways through which a medical history app may enhance patient satisfaction. Diagram created with Microsoft Powerpoint 2016. Image of patient holding a tablet generated with DALL·E using the prompt “oil painting of a female patient in a waiting room using a tablet for medical history taking” on March 7th, 2023-03-07

Based on literature about patient satisfaction, we can make assumptions about how the app may have enhanced satisfaction through several pathways. We illustrated these potential pathways in Fig. 4.

### Patient empowerment and patient engagement

The use of an app for symptom-oriented medical history can strengthen the patients’ sense of empowerment and sense of self-efficacy [34]. By providing all relevant health information in a structured format, the intervention may have shifted patients from passive to more active participants in their care. When patients actively document their health history, they may feel more engaged in their healthcare [35–37] and patient engagement may improve satisfaction [38, 39]. Thus, by encouraging reflection on symptoms, medication history, and concerns before the visit, the app may have promoted patient engagement.

### Preparation before consultation

A medical history app may help patients organize their thoughts [40]. This preparation could also reduce anxiety and increase confidence during the clinical encounter. Having thought about important details to cover during the consultation prior to it, patients may be able to articulate their concerns more clearly during the consultation. Therefore, patients might have perceived a more meaningful face-to-face time with physicians. In this study, we did not monitor physicians’ engagement with the app’s summary but we can assume that the app may have enabled more focused and efficient appointments. By organizing and presenting patient information clearly and concisely before the consultation, this may have allowed physicians a more targeted history-taking during the consultation, more time for explanation, reassurance, and shared decision-making – elements associated with patient satisfaction in primary care [41, 42] – rather than spending time on basic information gathering. This could lead to more focused and efficient consultations that felt more productive to patients.

### Feeling of being seen

Unlike traditional physician-led history taking, the app uses plain language instead of medical terminology. The app allows patients to respond to questions at their own pace. It eliminates the common problem of physicians interrupting patients after just 11–24 s [43]. In this context, the app might also make patients feel their full health story is accurately captured as the structured format ensures no important details are missed. As the information is transferred to their EMR, they get the impression that the physician is prepared for their interaction and physicians’ preparation for a clinical interaction fosters their connection with patients [44]. The app uses the same wording for everyone. This reduces potential bias related to gender, ethnicity, or cultural factors [40, 45, 46].

In sum, the pre-consultation medical history taking may have enhanced interactions by improving communication and leading to more focused and efficient consultations. This may have fostered patient satisfaction.

### Strengths and limitations

The study’s strength lies in its large sample size. The high response rate of 71% minimizes the risk of non-representative populations due to dropout. The surveys were handed out by study nurses and not by the physicians who could have excluded certain patients from the study (e.g. those with presumably negative attitudes). The cluster-randomized design strengthens causal inference compared to individual randomization, reducing contamination effects between intervention and control groups within the same urgent care clinics.

However, this study also comes with limitations. Despite a respectable participation rate, there is a potential selection bias as certain patient populations might have been more likely to take part in a study that entails engaging with an app. However, this applies for both control group and intervention group.

As we omitted questions from EUROPEP questionnaire that did not apply for the OOHP setting, this modified version is not validated. However, comparable minor former changes in the questionnaire did not impact its validity [47].

Data collection was performed in two centers. The differences show the importance of including more than one center and to consider different practice settings. However, they also suggest that it would have been fruitful to include more than two centers which was not possible due to COVID-19 pandemic-related travel restrictions.

The data collection during COVID-19 pandemic may have disproportionately impacted potential study participants and may limit our study’s generalizability. During pandemic, certain patient groups, e.g. vulnerable groups such as older people or those with multimorbid conditions, may have avoided seeing a physician or may have been more likely to refuse to participate in the study to avoid unnecessary contact.

During pandemic, patient organizational processes in the OOHP were partly reorganized. Waiting room and reception areas were redesigned (greater spacing between chairs, plexiglass screens at the counter and in the physicians’ rooms). These changes may have impacted patient satisfaction. However, these new workflows and furnishing were partly kept afterwards and, again, apply to both groups.

Our findings suggest that pre-consultation preparation tools can improve the patient experience. We discuss potential key mechanisms that converge to enhance overall patient satisfaction. However, based on our study we cannot conclude whether this framework explains the app’s effectiveness.

## Conclusion and future directions

We were able to show that the use of an app for medical history taking prior to medical consultations in OOHP increases patient satisfaction. Our results contribute to growing evidence that simple digital interventions can meaningfully impact care experiences. Unlike complex telemedicine platforms, medical history apps represent low-barrier digital tools with good usability [16, 48] that can be implemented across diverse healthcare settings. This scalability is important for health system adoption. For German healthcare specifically, our findings are relevant to ongoing digitalization efforts. The results suggest that patient-facing digital tools can complement rather than complicate existing urgent care workflows, potentially supporting both patient experience goals and operational efficiency.

Further research should focus on the mechanism how medical history taking by app enhances patient satisfaction. Furthermore, the impact on healthcare professionals’ satisfaction, which has been neglected in related research [31], should be examined as the success of the implementation of process innovations essentially depends on their experience. Another focus of future research could be the sustainability of app-based satisfaction improvements over time and if this results in (long-term) improvements of clinical outcomes (e.g. commitment to treatment, frequency of emergency room visits and hospitalizations).

## Supplementary Information

Below is the link to the electronic supplementary material.

Supplementary Material 1

Supplementary Material 2

Supplementary Material 3

Supplementary Material 4

Supplementary Material 5

## Data Availability

The data sets used and analyzed during the study are not publicly available due to the decision of the research ethics board but can be obtained from the authors upon reasonable request within a data sharing agreement.
