# Supplementary material for: Does an app make patients happy? Impact of a novel medical history app on patient satisfaction in urgent care consultations in Germany: cluster-randomized interventional trial ‘DASI’
Source: BMC Health Serv Res. 2026 May 29;26:771. doi: 10.1186/s12913-026-14795-6 (PMC13221757; doi:10.1186/s12913-026-14795-6)
Supplement: Supplementary file 1 — Supplementary Material 1 [file 12913_2026_14795_MOESM1_ESM.docx]

Additional file 1: EUROPEP items in English and German

|  | EUROPEP item in English | EUROPEP Item in German |
| --- | --- | --- |
| Nr. | What is your view on the GP with respect to…? | Wie hat der Arzt / die Ärztin... |
| 1 | …making you feel you had time during consultation? | ...Ihnen während des Besuchs das Gefühl vermittelt, dass er/sie Zeit für Sie hat? |
| 2 | …interest in your personal situation? | ...Interesse an Ihrer persönlichen Situation gezeigt? |
| 3 | …making it easy for you to tell him or her about your problems? | ...es Ihnen leicht gemacht, über Ihre Probleme zu sprechen? |
| 4 | …involving you in decisions about your medical care? | ...Sie in Entscheidungen über Ihre medizinische Behandlung einbezogen? |
| 5 | …listening to you? | ...Ihnen zugehört? |
| 6 | …thoroughness? | ...sich gründlich mit Ihren Problemen befasst? |
| 7 | …physical examination of you? | ...bei Ihnen körperliche Untersuchungen durchgeführt? |
| 8 | …explaining the purpose of tests and treatments? | ...Ihnen den Zweck von Untersuchungen und Behandlungen erklärt? |
| 9 | …explaining you advantages and disadvantages of treatment possibilities?* | ...Vor- und Nachteile unterschiedlicher Behandlungsmöglichkeiten erklärt? |
| 10 | …inform you about the effects and possible side effects of the medication he/she prescribed?* | ...Sie über Wirkung und mögliche Nebenwirkungen der von ihm/ihr verordneten Medikamente aufgeklärt? |
| 11 | …telling you what you wanted to know about your symptoms and/or illness? | ...Sie über das informiert, was Sie über Ihre Beschwerden bzw. Erkrankung wissen wollten? |
| 12 | …inform you about the pain you could expect during the examination and treatment? | ...Sie über zu erwartende Schmerzen bei Untersuchung und Therapie aufgeklärt? |
| 13 | …ask you about any pain?* | ...Sie nach eventuellen Schmerzen gefragt? |
| 14 | …helping you deal with emotional problems related to your health status? | ...Ihnen beim Umgang mit Ihren Gefühlen im Zusammenhang mit Ihrem Gesundheitszustand geholfen? |
| 15 | …helping you understand the importance of following his or her advice? | ...Ihnen erläutert, warum es wichtig ist, seine / ihre Ratschläge zu befolgen? |
| 16 | …inform you about what you can do yourself to heal/improve your symptoms (e.g. in everyday life)?* | ...Sie darüber informiert, was Sie selbst zur Heilung/ Besserung Ihrer Beschwerden beitragen können (z.B. im Alltag)? |
| 17 | …preparing you for what to expect from specialist or hospital care? | ...Sie darauf vorbereitet, was Sie bei anderen Ärzt*innen (bei Überweisungen) bzw. im Krankenhaus (bei Einweisungen) erwartet? |

Notes: The EUROPEP questionnaire may not be used without the consent of the aQua Institute. *Items translated into English by the authors.
