# Supplementary material for: Does an app make patients happy? Impact of a novel medical history app on patient satisfaction in urgent care consultations in Germany: cluster-randomized interventional trial ‘DASI’
Source: BMC Health Serv Res. 2026 May 29;26:771. doi: 10.1186/s12913-026-14795-6 (PMC13221757; doi:10.1186/s12913-026-14795-6)
Supplement: Supplementary file 2 — Supplementary Material 2 [file 12913_2026_14795_MOESM2_ESM.docx]

Additional file 3: Detailed results of EUROPEP - comparison between control and intervention group

| EUROPEP Item | |  | Control group  n = 538 | Intervention group  n = 496 |
| --- | --- | --- | --- | --- |
| Nr. | What is your view on the GP with respect to…? |  | n (%) | n (%) |
| 1 | …making you feel you had time during consultation? | 1 excellent | 372 (69.1) | 374 (75.4) |
|  |  | 2 | 119 (22.1) | 100 (20.2) |
|  |  | 3 | 31 (5.8) | 18 (3.6) |
|  |  | 4 | 11 (2.0) | 3 (0.6) |
|  |  | 5 poor | 4 (0.7) | 1 (0.2) |
|  |  | not applicable | 1 (0.2) | 0 (0.0) |
|  |  | no answer | 0 (0.0) | 0 (0.0) |
| 2 | …interest in your personal situation? | 1 excellent | 350 (65.1) | 345 (69.6) |
|  |  | 2 | 135 (25.1) | 118 (23.8) |
|  |  | 3 | 30 (5.6) | 22 (4.4) |
|  |  | 4 | 15 (2.8) | 6 (1.2) |
|  |  | 5 poor | 3 (0.6) | 2 (0.4) |
|  |  | not applicable | 3 (0.6) | 2 (0.4) |
|  |  | no answer | 2 (0.4) | 1 (0.2) |
| 3 | …making it easy for you to tell him or her about your problems? | 1 excellent | 381 (70.8) | 382 (77.0) |
|  |  | 2 | 113 (21.0) | 85 (17.1) |
|  |  | 3 | 32 (5.9) | 20 (4.0) |
|  |  | 4 | 5 (0.9) | 5 (1.0) |
|  |  | 5 poor | 3 (0.6) | 1 (0.2) |
|  |  | not applicable | 3 (0.6) | 3 (0.6) |
|  |  | no answer | 1 (0.2) | 0 (0.0) |
| 4 | …involving you in decisions about your medical care? | 1 excellent | 307 (57.1) | 328 (66.1) |
|  |  | 2 | 135 (25.1) | 118 (23.8) |
|  |  | 3 | 47 (8.7) | 20 (4.0) |
|  |  | 4 | 10 (1.9) | 9 (1.8) |
|  |  | 5 poor | 6 (1.1) | 3 (0.6) |
|  |  | not applicable | 31 (5.8) | 18 (3.6) |
|  |  | no answer | 2 (0.4) | 0 (0.0) |
| 5 | …listening to you? | 1 excellent | 415 (77.1) | 392 (79.0) |
|  |  | 2 | 92 (17.1) | 86 (17.3) |
|  |  | 3 | 20 ( 3.7) | 11 (2.2) |
|  |  | 4 | 6 ( 1.1) | 1 (0.2) |
|  |  | 5 poor | 2 ( 0.4) | 3 (0.6) |
|  |  | not applicable | 0 (0.0) | 0 (0.0) |
|  |  | no answer | 3 (0.6) | 3 (0.6) |
| 6 | …thoroughness? | 1 excellent | 330 (61.3) | 355 (71.6) |
|  |  | 2 | 143 (26.6) | 101 (20.4) |
|  |  | 3 | 34 (6.3) | 24 (4.8) |
|  |  | 4 | 21 (3.9) | 9 (1.8) |
|  |  | 5 poor | 4 (0.7) | 3 (0.6) |
|  |  | not applicable | 5 (0.9) | 4 (0.8) |
|  |  | no answer | 1 (0.2) | 0 (0.0) |
| 7 | …physical examination of you? | 1 excellent | 272 (50.6) | 299 (60.3) |
|  |  | 2 | 131 (24.3) | 97 (19.6) |
|  |  | 3 | 40 (7.4) | 33 (6.7) |
|  |  | 4 | 6 (1.1) | 6 (1.2) |
|  |  | 5 poor | 3 (0.6) | 3 (0.6) |
|  |  | not applicable | 86 (16.0) | 57 (11.5) |
|  |  | no answer | 0 (0.0) | 1 (0.2) |
| 8 | …explaining the purpose of tests and treatments? | 1 excellent | 263 (48.9) | 292 (58.9) |
|  |  | 2 | 130 (24.2) | 117 (23.6) |
|  |  | 3 | 47 (8.7) | 32 (6.5) |
|  |  | 4 | 17 (3.2) | 1 (0.2) |
|  |  | 5 poor | 5 (0.9) | 6 (1.2) |
|  |  | not applicable | 75 (13.9) | 46 (9.3) |
|  |  | no answer | 1 (0.2) | 2 (0.4) |
| 9 | …explaining you advantages and disadvantages of treatment possibilities? | 1 excellent | 181 (33.6) | 210 (42.3) |
|  |  | 2 | 102 (19.0) | 89 (17.9) |
|  |  | 3 | 56 (10.4) | 45 (9.1) |
|  |  | 4 | 19 (3.5) | 7 (1.4) |
|  |  | 5 poor | 7 (1.3) | 6 (1.2) |
|  |  | not applicable | 172 (32.0) | 137 (27.6) |
|  |  | no answer | 1 (0.2) | 2 (0.4) |
| 10 | …inform you about the effects and possible side effects of the medication he/she prescribed? | 1 excellent | 160 (29.7) | 188 (37.9) |
|  |  | 2 | 89 (16.5) | 90 (18.1) |
|  |  | 3 | 60 (11.2) | 36 (7.3) |
|  |  | 4 | 19 (3.5) | 14 (2.8) |
|  |  | 5 poor | 15 (2.8) | 5 (1.0) |
|  |  | not applicable | 192 (35.7) | 161 (32.5) |
|  |  | no answer | 3 (0.6) | 2 (0.4) |
| 11 | …telling you what you wanted to know about your symptoms and/or illness? | 1 excellent | 285 (53.0) | 294 (59.3) |
|  |  | 2 | 135 (25.1) | 110 (22.2) |
|  |  | 3 | 40 (7.4) | 27 (5.4) |
|  |  | 4 | 11 (2.0) | 11 (2.2) |
|  |  | 5 poor | 7 (1.3) | 4 (0.8) |
|  |  | not applicable | 60 (11.2) | 47 (9.5) |
|  |  | no answer | 0 (0.0) | 3 (0.6) |
| 12 | …inform you about the pain you could expect during the examination and treatment? | 1 excellent | 193 (35.9) | 196 (39.5) |
|  |  | 2 | 94 (17.5) | 77 (15.5) |
|  |  | 3 | 29 (5.4) | 29 (5.8) |
|  |  | 4 | 13 (2.4) | 9 (1.8) |
|  |  | 5 poor | 9 (1.7) | 1 (0.2) |
|  |  | not applicable | 199 (37.0) | 181 (36.5) |
|  |  | no answer | 1 (0.2) | 3 (0.6) |
| 13 | …ask you about any pain? | 1 excellent | 351 (65.2) | 373 (75.2) |
|  |  | 2 | 100 (18.6) | 75 (15.1) |
|  |  | 3 | 26 (4.8) | 15 (3.0) |
|  |  | 4 | 16 (3.0) | 10 (2.0) |
|  |  | 5 poor | 8 (1.5) | 4 (0.8) |
|  |  | not applicable | 36 (6.7) | 17 (3.4) |
|  |  | no answer | 1 (0.2) | 2 (0.4) |
| 14 | …helping you deal with emotional problems related to your health status? | 1 excellent | 232 (43.1) | 257 (51.8) |
|  |  | 2 | 108 (20.1) | 104 (21.0) |
|  |  | 3 | 37 (6.9) | 27 (5.4) |
|  |  | 4 | 26 (4.8) | 15 (3.0) |
|  |  | 5 poor | 9 (1.7) | 5 (1.0) |
|  |  | not applicable | 125 (23.2) | 83 (16.7) |
|  |  | no answer | 1 (0.2) | 5 (1.0) |
| 15 | …helping you understand the importance of following his or her advice? | 1 excellent | 230 (42.8) | 235 (47.4) |
|  |  | 2 | 93 (17.3) | 96 (19.4) |
|  |  | 3 | 50 (9.3) | 37 (7.5) |
|  |  | 4 | 16 (3.0) | 6 (1.2) |
|  |  | 5 poor | 8 (1.5) | 4 (0.8) |
|  |  | not applicable | 140 (26.0) | 114 (23.0) |
|  |  | no answer | 1 (0.2) | 4 (0.8) |
| 16 | …inform you about what you can do yourself to heal/improve your symptoms (e.g. in everyday life)? | 1 excellent | 266 (49.4) | 265 (53.4) |
|  |  | 2 | 107 (19.9) | 86 (17.3) |
|  |  | 3 | 38 (7.1) | 23 (4.6) |
|  |  | 4 | 16 (3.0) | 9 (1.8) |
|  |  | 5 poor | 13 (2.4) | 10 (2.0) |
|  |  | not applicable | 97 (18.0) | 97 (19.6) |
|  |  | no answer | 1 (0.2) | 6 (1.2) |
| 17 | …preparing you for what to expect from specialist or hospital care? | 1 excellent | 174 (32.3) | 188 (37.9) |
|  |  | 2 | 89 (16.5) | 76 (15.3) |
|  |  | 3 | 30 (5.6) | 27 (5.4) |
|  |  | 4 | 8 (1.5) | 4 (0.8) |
|  |  | 5 poor | 7 (1.3) | 3 (0.6) |
|  |  | not applicable | 228 (42.4) | 191 (38.5) |
|  |  | no answer | 2 (0.4) | 7 (1.4) |
