# Supplementary material for: Does an app make patients happy? Impact of a novel medical history app on patient satisfaction in urgent care consultations in Germany: cluster-randomized interventional trial ‘DASI’
Source: BMC Health Serv Res. 2026 May 29;26:771. doi: 10.1186/s12913-026-14795-6 (PMC13221757; doi:10.1186/s12913-026-14795-6)
Supplement: Supplementary file 3 — Supplementary Material 3 [file 12913_2026_14795_MOESM3_ESM.docx]

Additional file 2: Characteristics of participants - comparison between centers

|  |  | **Overall**  **n = 1,034** | **Göttingen**  **n = 573** | **Northeim**  **n = 461** | **p** |
| --- | --- | --- | --- | --- | --- |
| Sex, n (%) | male | 410 (40%) | 231 (40%) | 179 (39%) | 0.627 |
|  | female | 624 (60%) | 342 (60%) | 282 (61%) |  |
| Age, median (IQR) | years | 31 (24, 44) | 29 (23, 39) | 34 (26, 50) | **<0.001** |
| Number of complaints selected in the app, median (IQR) | n | 1 (1, 2) | 1 (1, 2) | 1 (1, 2) | 0.081 |
| Severity of complaints, n (%) | I don’t feel sick | 112 (11%) | 64 (11%) | 48 (10%) | 0.892 |
|  | Mild | 192 (19%) | 106 (19%) | 86 (19%) |  |
|  | Medium | 512 (50%) | 280 (49%) | 232 (50%) |  |
|  | Severe | 195 (19%) | 109 (19%) | 86 (19%) |  |
|  | Unbearable | 20 (1.9%) | 11 (1.9%) | 9 (2.0%) |  |
| Highest professional qualification, n (%) | Master’s degree/Diploma/State examination/ PhD | 140 (14%) | 98 (17%) | 42 (9.2%) | **<0.001** |
|  | Bachelor‘s degree | 82 (8.0%) | 63 (11%) | 19 (4.1%) |  |
|  | Master craftsman/technician or equivalent | 42 (4.1%) | 20 (3.5%) | 22 (4.8%) |  |
|  | Completed vocational training | 248 (24%) | 115 (20%) | 133 (29%) |  |
|  | High school diploma / Advanced technical college certificate | 224 (22%) | 153 (27%) | 71 (16%) |  |
|  | Secondary school certificate | 177 (17%) | 75 (13%) | 102 (22%) |  |
|  | Elementary/lower secondary school certificate | 88 (8.6%) | 30 (5.3%) | 58 (13%) |  |
|  | Other qualifications | 14 (1.4%) | 9 (1.6%) | 5 (1.1%) |  |
|  | No degree | 14 (1.4%) | 8 (1.4%) | 6 (1.3%) |  |
| Current employment status, n (%) | Employed/working | 563 (56%) | 281 (50%) | 282 (63%) | **<0.001** |
|  | In vocational training | 85 (8.4%) | 42 (7.5%) | 43 (9.6%) |  |
|  | University student | 160 (16%) | 150 (27%) | 10 (2.2%) |  |
|  | Voluntary/civilian/military service | 6 (0.6%) | 5 (0.9%) | 1 (0.2%) |  |
|  | Student at school | 24 (2.4%) | 11 (2.0%) | 13 (2.9%) |  |
|  | Retired | 63 (6.2%) | 21 (3.7%) | 42 (9.4%) |  |
|  | Unemployed | 20 (2.0%) | 10 (1.8%) | 10 (2.2%) |  |
|  | other | 88 (8.7%) | 41 (7.3%) | 47 (10%) |  |
| German as native language, n (%) | Yes | 906 (88%) | 492 (86%) | 414 (91%) | **0.029** |
| Study group, n (%) | Intervention group | 496 (48%) | 262 (46%) | 234 (51%) | 0.107 |

P-values < 0.05 in bold (Mann-Whitney U-Test for continuous variables, Chi-square test for categorical variables, Mann–Whitney U test for ordinal variables).
