# Supplementary material for: Does an app make patients happy? Impact of a novel medical history app on patient satisfaction in urgent care consultations in Germany: cluster-randomized interventional trial ‘DASI’
Source: BMC Health Serv Res. 2026 May 29;26:771. doi: 10.1186/s12913-026-14795-6 (PMC13221757; doi:10.1186/s12913-026-14795-6)
Supplement: Supplementary file 4 — Supplementary Material 4 [file 12913_2026_14795_MOESM4_ESM.docx]

Additional file 5: EUROPEP domain scores

| **Subscore** | **Group** | **M** | **Lower bound of CI** | **Higher bound of CI** |
| --- | --- | --- | --- | --- |
| Relation and communication | control group | 1.424 | 1.373 | 1.475 |
| Relation and communication | intervention group | 1.331 | 1.284 | 1.377 |
| Medical care | control group | 1.576 | 1.513 | 1.64 |
| Medical care | intervention group | 1.421 | 1.364 | 1.477 |
| Information and support | control group | 1.749 | 1.679 | 1.819 |
| Information and support | intervention group | 1.576 | 1.511 | 1.64 |

*M* arithmetic mean. CI: confidence interval. Lower scores indicate higher satisfaction.

|  | ***M*** | **Lower bound of CI** | **Higher bound of CI** | **p (adj)*** |
| --- | --- | --- | --- | --- |
| Relation and communication | 1.379 | 1.344 | 1.414 | **0.003** |
| Medical care | 1.502 | 1.459 | 1.545 | **<0.001** |
| Information and support | 1.666 | 1.618 | 1.714 | **<0.001** |

*M* arithmetic mean. CI: confidence interval. P-values < 0.05 in bold (comparison between intervention and control group). Higher scores indicate higher satisfaction scores in intervention compared to control group. *p (adjusted) for severity of complaints and time clusters
