# Supplementary material for: Does an app make patients happy? Impact of a novel medical history app on patient satisfaction in urgent care consultations in Germany: cluster-randomized interventional trial ‘DASI’
Source: BMC Health Serv Res. 2026 May 29;26:771. doi: 10.1186/s12913-026-14795-6 (PMC13221757; doi:10.1186/s12913-026-14795-6)
Supplement: Supplementary file 5 — Supplementary Material 5 [file 12913_2026_14795_MOESM5_ESM.docx]

Additional file 4: EUROPEP – comparison between centers

| EUROPEP Item | |  | Overall  N = 1,034 | | Göttingen  n = 573 | | Northeim  n = 461 | |  |
| --- | --- | --- | --- | --- | --- | --- | --- | --- | --- |
| Nr. | What is your view on the GP with respect to…? | n | Median (IQR) | *M* (SD) | Median (IQR) | *M* (SD) | Median (IQR) | *M* (SD) | p (adj)* |
| 1 | …making you feel you had time during consultation? | 1,033 | 1.00 (1.00, 2.00) | 1.37 (0.68) | 1.00 (1.00, 2.00) | 1.45 (0.75) | 1.00 (1.00, 1.00) | 1.26 (0.57) | **<0.001** |
| 2 | …interest in your personal situation? | 1,026 | 1.00 (1.00, 2.00) | 1.43 (0.73) | 1.00 (1.00, 2.00) | 1.48 (0.79) | 1.00 (1.00, 2.00) | 1.36 (0.63) | **0.009** |
| 3 | …making it easy for you to tell him or her about your problems? | 1,027 | 1.00 (1.00, 2.00) | 1.34 (0.66) | 1.00 (1.00, 2.00) | 1.38 (0.70) | 1.00 (1.00, 1.00) | 1.28 (0.60) | **0.017** |
| 4 | …involving you in decisions about your medical care? | 983 | 1.00 (1.00, 2.00) | 1.49 (0.78) | 1.00 (1.00, 2.00) | 1.59 (0.89) | 1.00 (1.00, 2.00) | 1.37 (0.62) | **<0.001** |
| 5 | …listening to you? | 1,028 | 1.00 (1.00, 1.00) | 1.27 (0.60) | 1.00 (1.00, 1.00) | 1.31 (0.64) | 1.00 (1.00, 1.00) | 1.23 (0.54) | 0.053 |
| 6 | …thoroughness? | 1,024 | 1.00 (1.00, 2.00) | 1.47 (0.79) | 1.00 (1.00, 2.00) | 1.58 (0.87) | 1.00 (1.00, 2.00) | 1.33 (0.66) | **<0.001** |
| 7 | …physical examination of you? | 890 | 1.00 (1.00, 2.00) | 1.49 (0.76) | 1.00 (1.00, 2.00) | 1.53 (0.83) | 1.00 (1.00, 2.00) | 1.44 (0.67) | 0.071 |
| 8 | …explaining the purpose of tests and treatments? | 910 | 1.00 (1.00, 2.00) | 1.55 (0.83) | 1.00 (1.00, 2.00) | 1.66 (0.92) | 1.00 (1.00, 2.00) | 1.43 (0.69) | **<0.001** |
| 9 | …explaining you advantages and disadvantages of treatment possibilities? | 722 | 1.00 (1.00, 2.00) | 1.72 (0.96) | 1.00 (1.00, 2.00) | 1.81 (1.00) | 1.00 (1.00, 2.00) | 1.63 (0.90) | **0.01** |
| 10 | …inform you about the effects and possible side effects of the medication he/she prescribed? | 676 | 1.00 (1.00, 2.00) | 1.81 (1.04) | 2.00 (1.00, 3.00) | 1.97 (1.14) | 1.00 (1.00, 2.00) | 1.65 (0.91) | **<0.001** |
| 11 | …telling you what you wanted to know about your symptoms and/or illness? | 924 | 1.00 (1.00, 2.00) | 1.53 (0.82) | 1.00 (1.00, 2.00) | 1.61 (0.90) | 1.00 (1.00, 2.00) | 1.43 (0.72) | **0.001** |
| 12 | …inform you about the pain you could expect during the examination and treatment? | 650 | 1.00 (1.00, 2.00) | 1.60 (0.90) | 1.00 (1.00, 2.00) | 1.68 (0.99) | 1.00 (1.00, 2.00) | 1.52 (0.79) | **0.027** |
| 13 | …ask you about any pain? | 978 | 1.00 (1.00, 2.00) | 1.39 (0.79) | 1.00 (1.00, 2.00) | 1.49 (0.90) | 1.00 (1.00, 1.00) | 1.28 (0.62) | **<0.001** |
| 14 | …helping you deal with emotional problems related to your health status? | 820 | 1.00 (1.00, 2.00) | 1.63 (0.95) | 1.00 (1.00, 2.00) | 1.71 (1.04) | 1.00 (1.00, 2.00) | 1.54 (0.82) | **0.011** |
| 15 | …helping you understand the importance of following his or her advice? | 775 | 1.00 (1.00, 2.00) | 1.62 (0.90) | 1.00 (1.00, 2.00) | 1.72 (0.99) | 1.00 (1.00, 2.00) | 1.50 (0.78) | **0.001** |
| 16 | …inform you about what you can do yourself to heal/improve your symptoms (e.g. in everyday life)? | 833 | 1.00 (1.00, 2.00) | 1.58 (0.95) | 1.00 (1.00, 2.00) | 1.72 (1.08) | 1.00 (1.00, 2.00) | 1.42 (0.75) | **<0.001** |
| 17 | …preparing you for what to expect from specialist or hospital care? | 606 | 1.00 (1.00, 2.00) | 1.59 (0.87) | 1.00 (1.00, 2.00) | 1.65 (0.89) | 1.00 (1.00, 2.00) | 1.52 (0.84) | 0.054 |

Remarks: Items are scored 1–5 where higher scores represent better experiences. The response scale for all items was a five-point scale with the endpoints very dissatisfied and very satisfied. IQR: interquartile range; *M* arithmetic mean. P-values < 0.05 in bold. *p (adjusted) for severity of complaints and time clusters.

EUROPEP – comparison between centers (subgroup analysis, excluding university students)

| EUROPEP Item | |  | Overall  N = 761 | | Göttingen  n = 370 | | Northeim  n = 391 | |  |
| --- | --- | --- | --- | --- | --- | --- | --- | --- | --- |
| Nr. | What is your view on the GP with respect to…? | n | Median (IQR) | *M* (SD) | Median (IQR) | *M* (SD) | Median (IQR) | *M* (SD) | p (adj)* |
| 1 | …making you feel you had time during consultation? | 760 | 1.00 (1.00, 2.00) | 1.34 (0.66) | 1.00 (1.00, 2.00) | 1.42 (0.74) | 1.00 (1.00, 1.00) | 1.27 (0.57) | **0.002** |
| 2 | …interest in your personal situation? | 755 | 1.00 (1.00, 2.00) | 1.42 (0.71) | 1.00 (1.00, 2.00) | 1.46 (0.77) | 1.00 (1.00, 2.00) | 1.39 (0.65) | 0.136 |
| 3 | …making it easy for you to tell him or her about your problems? | 755 | 1.00 (1.00, 2.00) | 1.32 (0.64) | 1.00 (1.00, 2.00) | 1.36 (0.67) | 1.00 (1.00, 1.00) | 1.29 (0.61) | 0.15 |
| 4 | …involving you in decisions about your medical care? | 728 | 1.00 (1.00, 2.00) | 1.46 (0.74) | 1.00 (1.00, 2.00) | 1.53 (0.83) | 1.00 (1.00, 2.00) | 1.40 (0.64) | **0.013** |
| 5 | …listening to you? | 758 | 1.00 (1.00, 1.00) | 1.27 (0.59) | 1.00 (1.00, 1.00) | 1.29 (0.61) | 1.00 (1.00, 1.00) | 1.25 (0.56) | 0.325 |
| 6 | …thoroughness? | 752 | 1.00 (1.00, 2.00) | 1.43 (0.76) | 1.00 (1.00, 2.00) | 1.51 (0.83) | 1.00 (1.00, 2.00) | 1.35 (0.69) | **0.004** |
| 7 | …physical examination of you? | 662 | 1.00 (1.00, 2.00) | 1.50 (0.76) | 1.00 (1.00, 2.00) | 1.52 (0.84) | 1.00 (1.00, 2.00) | 1.48 (0.69) | 0.543 |
| 8 | …explaining the purpose of tests and treatments? | 676 | 1.00 (1.00, 2.00) | 1.52 (0.80) | 1.00 (1.00, 2.00) | 1.59 (0.88) | 1.00 (1.00, 2.00) | 1.46 (0.72) | **0.044** |
| 9 | …explaining you advantages and disadvantages of treatment possibilities? | 532 | 1.00 (1.00, 2.00) | 1.71 (0.94) | 1.00 (1.00, 2.00) | 1.74 (0.95) | 1.00 (1.00, 2.00) | 1.69 (0.93) | 0.589 |
| 10 | …inform you about the effects and possible side effects of the medication he/she prescribed? | 507 | 1.00 (1.00, 2.00) | 1.80 (1.01) | 2.00 (1.00, 3.00) | 1.90 (1.10) | 1.00 (1.00, 2.00) | 1.72 (0.93) | 0.053 |
| 11 | …telling you what you wanted to know about your symptoms and/or illness? | 680 | 1.00 (1.00, 2.00) | 1.51 (0.79) | 1.00 (1.00, 2.00) | 1.55 (0.83) | 1.00 (1.00, 2.00) | 1.47 (0.74) | 0.186 |
| 12 | …inform you about the pain you could expect during the examination and treatment? | 485 | 1.00 (1.00, 2.00) | 1.59 (0.86) | 1.00 (1.00, 2.00) | 1.60 (0.92) | 1.00 (1.00, 2.00) | 1.58 (0.82) | 0.763 |
| 13 | …ask you about any pain? | 720 | 1.00 (1.00, 1.00) | 1.37 (0.77) | 1.00 (1.00, 2.00) | 1.45 (0.87) | 1.00 (1.00, 1.00) | 1.30 (0.65) | **0.006** |
| 14 | …helping you deal with emotional problems related to your health status? | 598 | 1.00 (1.00, 2.00) | 1.61 (0.90) | 1.00 (1.00, 2.00) | 1.61 (0.95) | 1.00 (1.00, 2.00) | 1.60 (0.85) | 0.829 |
| 15 | …helping you understand the importance of following his or her advice? | 573 | 1.00 (1.00, 2.00) | 1.59 (0.85) | 1.00 (1.00, 2.00) | 1.64 (0.91) | 1.00 (1.00, 2.00) | 1.55 (0.80) | 0.252 |
| 16 | …inform you about what you can do yourself to heal/improve your symptoms (e.g. in everyday life)? | 613 | 1.00 (1.00, 2.00) | 1.54 (0.88) | 1.00 (1.00, 2.00) | 1.65 (1.00) | 1.00 (1.00, 2.00) | 1.45 (0.76) | **0.006** |
| 17 | …preparing you for what to expect from specialist or hospital care? | 433 | 1.00 (1.00, 2.00) | 1.60 (0.88) | 1.00 (1.00, 2.00) | 1.63 (0.89) | 1.00 (1.00, 2.00) | 1.58 (0.88) | 0.546 |

Remarks: Items are scored 1–5 where higher scores represent better experiences. The response scale for all items was a five-point scale with the endpoints very dissatisfied and very satisfied. IQR: interquartile range; *M* arithmetic mean. P-values < 0.05 in bold. *p (adjusted) for severity of complaints and time clusters.
